# Malaria and helminth co-infections in outpatients of Alaba Kulito Health Center, southern Ethiopia: a cross sectional study

**DOI:** 10.1186/1756-0500-3-143

**Published:** 2010-05-25

**Authors:** Abraham Degarege, Abebe Animut, Mengistu Legesse, Berhanu Erko

**Affiliations:** 1 Bahir Dar University, PO Box 79, Bahir Dar, Ethiopia; 2 Aklilu Lemma Institute of Pathobiology, Addis Ababa University, PO Box 1176, Addis Ababa, Ethiopia

## Abstract

**Background:**

Distribution of malaria and intestinal helminths is known to overlap in developing tropical countries of the world. Co-infections with helminth and malaria parasites cause a significant and additive problem against the host. The aim of this study was to asses the prevalence of malaria/helminth co-infection and the associated problems among febrile outpatients that attended Alaba Kulito Health Center, southern Ethiopia November and December 2007. A total of 1802 acute febrile patients were diagnosed for malaria. 458 Giemsa-stained thick and thin blood films were used for identification of *Plasmodium *species and Stool samples prepared using Kato-Katz technique were used to examine for intestinal helminths. Haemoglobin concentration was measured using a portable spectrophotometer (Hemocue HB 201). Anthropometry-based nutritional assessment of the study participants was done by measuring body weight to the nearest 0.1 kg and height to the nearest 0.1 cm.

**Findings:**

458 of the total febrile patients were positive for malaria. Co infection with *Plasmodium *and helminth parasites is associated with significantly (p < 0.001) higher anaemia prevalence than single infection with *Plasmodium *parasites. And this difference was also significant for haemoglobin concentration (F = 10.18, p = 0.002), in which patients co infected with *Plasmodium *and helminth parasites showed lower mean haemoglobin concentration. More than one-third of the infected cases in both malaria infections and malaria/helminth co infections are undernourished. However the statistics for the difference is not significant.

**Conclusion:**

Malaria and soil-transmitted helminthiasis obviously contribute to anaemia and low weight status and these conditions are more pronounced in individuals concurrently infected with malaria and soil-transmitted helminths. Hence, simultaneous combat against the two parasitic infections is very crucial to improve health of the affected communities.

## Background

Overlapping distribution of intestinal helminths and malaria results in a high rate of co-infection [1,2], which may result both in synergism and antagonistic interaction between helminths and malaria parasites [3,4]. One of the main impacts of malaria and helminth infections is anaemia. Malaria causes anaemia, among other mechanisms through haemolysis and increased spleenic clearance of infected and uninfected red blood cells and cytokine-induced dyserythropoeisis [5,6]. Similarly, intestinal helminths are significant causes of anaemia as a result of direct blood loss, nutritional theft and impairment of the appetite due to immunological factor [7,8]. Based on the distinct mechanisms by which malaria and helminths reduce haemoglobin levels, it can be speculated that their combined presence might interact to enhance the risk of anaemia. And several reports [9-12] in Kenya, Nigeria, Thailand and some other countries of Africa showed suggestive of an additive impact of co-infection on anaemia in certain age groups. However such associations may be confounded by socio-economic, genetic, and nutritional factors and that the effects of co-infection may vary by malaria and helminth species and their intensities [10].

In addition, co-infections with helminth and malaria parasites have negative impact upon host nutrition through a number of mechanisms which may have additive or multiplicative impacts, especially in childhood [13]. Helminths cause and/or aggravate malnutrition through worm-induced gastrointestinal tract physiopathology and reduced food intake [14], chronic blood-loss [8] and intestinal inflammation [15]. Malaria may also contribute to protein-energy malnutrition through a number of mechanisms triggered by augmented levels of inflammatory cytokines, including anorexia and the induction of a catabolic response [16]. However, the contribution of intestinal helminth infections as well as malaria in the development of anaemia and low weight status, the concomitant occurrence of malaria and intestinal helminth infections and their clinical manifestations among all age groups, in malaria endemic areas like Ethiopia is largely unreported. Therefore, the aims of this study were to asses the associations between anemia/nutrition status and helminth infections in patients with clinical malaria in communities of Alaba Kulito area, southern Ethiopia.

## Methods

### Study area and population

The study was conducted at Alaba Kulito Health Center in Alaba Woreda (administrative unit), located 313 km south of Addis Ababa. The Woreda is semi-arid with annual temperature ranging from 18 to 23°C and mean annual rainfall from 100 to 120 millimeter. Malaria and intestinal parasites are the most prevalent public health problems in the area. Malaria transmission in Alaba is unstable, seasonal and depends on altitude and rainfall. There are two main seasons for transmission of the disease; September to December, after the heavy summer rains, and March to May, after the light rains.

The study was conducted on acute malaria patients that attended Alaba Kulito Health Center in November and December 2007. Cases positive for *Plasmodium *species and older than one year, had no history of anti-malarial drug administration in the two weeks prior to screening, absence of any other serious chronic infection, had ability to give blood and stool samples were included in the study. However, pregnant women, children younger than 1 year and individuals with known concomitant chronic infection were excluded from the study, and this work has been published elsewhere [17].

### Nutritional assessment

Anthropometry-based nutritional assessment of the study participants was done by measuring body weight to the nearest 0.1 kg and height to the nearest 0.1 cm. Then the body mass index (BMI) was calculated to determine the weight status of the study participants using the formula (weight in kg/[height in m]^2^). In classifying the weight status of the study participants the standard weight status categories associated with BMI ranges were used for overweight and obesity in adult [18]. Similarly, the international BMI cut offs for child overweight and obesity which cover the age range 1-19 years were used for children and adolescents [18,19]. However, the interpretation of BMI values for age group 1-19 years was done after obtaining a percentile rank using a CDC-BM/-For age growth charts and Statistical Advanced Soft Ware [20] which convert BMI values to an ordered grade interpolating to the child's age. In addition, for underweight (thinness) three cut offs, namely grade-1 thinness (BMI < 16 Kg/m^2^), grade-2 thinness (BMI = 16-16.9 Kg/m^2^) and grade-3 thinness (BMI = 17-18.4 kg/m^2^) were used [18-20] in case of adults. For children and adolescents similar cut off values derived based on adults BMI 16 and 16.5 at 18 years were used [21].

### Clinical and laboratory diagnosis

Socio-demographic survey and clinical diagnosis were made by trained physicians of the health center. Thick and thin smears were prepared on a single slide for each acute febrile patient from capillary blood by finger pricking using sterile lancet. Each blood smear was stained with Giemsa and examined under the oil immersion microscope objective. Hundred fields were examined before negative result was reported. Thick smear was used to detect malaria infection and parasite quantification. The thin smear was used to identify the type of *Plasmodium *species. The number of parasites per microliter of blood was calculated from the thick blood smear [22]. For each participant only one slide was read to detect malaria infection and identify the type of *Plasmodium *species.

Clean stool cap and stick applicator were provided to malaria positive subjects volunteered to provide stool specimen. Kato-Katz thick method was used to prepare stool specimens for microscopic examination [23]. Similarly, for each participant only one slide was read to detect helminth infection and identify the type of species.

### Determination of haemoglobin concentration

Haemoglobin concentration was determined using a portable haemoglobin spectrophotometer, Hemocue Hb 201 analyzer (HemoCue, Angelholm, Sweden) and specially designed microcuvette (the Hemocue Hb 201 Microcuvette, Hemocue, Angelholm, Sweden). Then, the haemoglobin values were used to assess the status of anaemia. For haemoglobin, the cut-off criterion levels below which indicating anemia was the WHO cut-off of 110 g/L for children 6-59 months; 115 g/L for children 5-11 years; 120 g/L for children 12-14 years; 120 g/L for non-pregnant women above 15 years of age and 130 g/L for men above 15 years of age [24].

### Data analysis

Data entry and validation was performed in excel, and statistical analysis was done using Statistical Package for Social Science version 13.0. Mantel Heinzel Chi-square test including odd ratios at 95% CI and one way ANOVA were used to test for differences in proportions and means, respectively. Values were considered statistically significant when p-values were less than 0.05.

### Ethical consideration

This study was part of the ongoing project on efficacy of anti-malarial drugs against malaria at the Akililu Lemma Institute of Pathobiology, Addis Ababa University. The study was conducted after ethical clearance was obtained from the Institute and the Health Bureau of South Nations Nationalities and Peoples Region (SNNPR). Patients were involved in the study after obtaining informed consent. Consent was also obtained from guardians and/or parents for children under 18 years of age. All positive subjects for *Plasmodium falciparum *were treated with coartem, for *P.vivax *with chloroquine, for mixed infection with *P. falciparum *and *P. vivax *with coartem and chloroquine and for soil-transmitted helminthiasis with albendazole.

## Result

### Malaria and soil-transmitted helminth infections

Out of the 1802 acute febrile patients, 502 (27.9%) were positive for *Plasmodium *parasites and 458 subjects (233 males and 225 females) fulfilled the inclusion criteria and enrolled in the co-infection study. From the 458 malaria infected patients, 255 (55.7%) were positive for one or more STHs. The most prevalent helminth was hookworm (37.8%) followed by *A. lumbricoides *(24.7%) and *T. trichiura *(8.3%) [17].

The overall intensity of infection of intestinal helminthiasis expressed as geometric mean among the study subjects for children younger than 5 years of age, children 5-14 years old and adults ≥ 15 years old was, 955.33 (range; 24-7680), 2309 (range; 96-64424) and 1045.8(range; 24-30248), respectively. The intensity of infection tends to reach its peak in the age group ≥ 15 years old for hookworm, 5-14 years old for *A. lumbricoides *and < 5 years old for *T. trichiura *with a mean EPG of 542.72, 6249.9 and 699.33, respectively.

### Haemoglobin measurement

Mean haemoglobin concentration of the study participants was, 13.1 g/dl (ranging from 5.2 g/dl to 21.4 g/dl) with standard deviation of 2.37. *Plasmodium *alone or both *Plasmodium *and helminth infected females were found to have significantly lower mean haemoglobin concentration than males. Children younger than 5 years were found to have significantly the lowest mean haemoglobin concentration as compared to the older age groups.

Based on the WHO cut off values of haemoglobin concentration, a total of 144 anaemic cases were found, making an overall prevalence of anaemia among the study subjects 31.4%. Severe anaemia (haemoglobin concentration ≤ 7 g/dl) was very rarely seen (1.7%) and most of these were young children.

Prevalence of anaemia was higher in females (33.3%) than in males (29.6%) and highest in children younger than 5 years (46.3%) (p = 0.003). It was observed that 18.7%, 40.7% and 50% of *P. vivax, P. falciparum *and mixed infected subjects were anaemic, respectively. Similarly, the prevalence of anaemia was higher in malaria and intestinal helminth co-infected patients. The prevalence of anaemia was 43.3%, 29.2%, 35.7% and 42.3% for hookworm, *A. lumbricoides*, *T. trichiura *and multiple intestinal helminth and malaria co infected subjects, respectively (Table 1). In general, data for the study patients indicate that co infection with *Plasmodium *and helminth parasites is associated with significantly (x^2 ^= 20.3, Odds Ratio = 2.58, p < 0.001) higher anaemia prevalence than single infection with *Plasmodium *parasites (Table 2). And this difference was also significant for haemoglobin concentration (F = 10.18, p = 0.002), in which patients co infected with *Plasmodium *and helminth parasites showed lower mean haemoglobin concentration.

**Table 1:** Prevalence of anaemia by infection types among the study participants at Alaba Kulito Health Center, SNNPR, South Ethiopia, Nov-Dec, 2007

Variables	Anaemia N (%)	Non-anaemia N (%)	Statistics
Malaria infection*			
Pv	31(18.7)	135(81.3)	X^2 ^= 10. 6
Pf	11(40.7)	16(59.3)	P = 0.005
Pv & Pf	5(50)	5(50)	

Co-infection			
Malaria/Hw	45(43.3)	59(53.7)	X^2 ^= 3.02
Malaria/Al	14(29.2)	34(70.8)	P = 0.388
Malaria/Tt	5(35.7)	9(64.3)	
Malaria/mih	33(42.3)	45(57.7)	

* Malaria infection without concurrent intestinal helminth infection mih = multiple (two or more) intestinal helminth infection

**Table 2:** Percent prevalence of anaemia in different age groups and sexes (Comparison between cases with malaria infections and malaria/Helminth co infections in all age and sex groups) Alaba Kulito Health Center, SNNPR, South Ethiopia, Nov-Dec, 2007

Age	% Co-infected cases who were anaemic(n/N)	%Malaria infection*cases Who were anaemic (n/N)	Adjusted** Odds Ratio,(95%CI), x^2^(df), p-value	Over all Adjusted***Odds Ratio,(95%CI), x^2^(df), p-value
< 5	72 (18/25)	31 (13/42)	5.74(1.93-17.08) 10.5(1),0.001	2.58(1.7-3.92) 20.3(1), p < 0.001
5-14	39 (23/59)	30 (9/30)	1.49(0.58-3.82) 0.7(1),0.416	
≥ 15	36.2(59/163)	15.8(22/139)	3.02(1.73-5.26) 15.8(1), p < 0.001	

Sex				
F	35.7 (46/129)	30.2 (29/96)	1.28(0.73-2.25) 0.7(1),0.392	2.58(1.7-3.92) 20.3(1), p < 0.001
M	45.8 (54/118)	13 (15/115)	5.62(2.93-10.8) 29.8(1), p < 0.001	

* Malaria infection without concurrent intestinal helminth infection mih = multiple (two or more) intestinal helminth infection

** The age-specific ratios within the cases were adjusted for sex or age

*** Within each cases the over all Odds were adjusted for both age and sex. For the combined data the odds were adjusted for cases, age and sex

Co infection with *Plasmodium *and helminth parasites was significantly (p ≤ 0.001) associated with high prevalence of anaemia than single infection with *Plasmodium *parasites in children younger than 5 years and adults ≥ 15 years. And this difference was also significant for haemoglobin concentration (F = 18.17, p < 0.001), in which patients co infected with *Plasmodium *and helminth parasites showed lower mean haemoglobin concentration. Similarly, the prevalence of anaemia in males was higher (p < 0.001) in malaria and intestinal helminth co infected patients than those single infected with malaria. In contrast, no significance difference was observed both among females and children, 5-14 years (Table 2).

### Nutritional assessment

The BMI of the study participants calculated from their body weight and height measures revealed that 19.0% (87/458), 5.9% (27/458), 13.5% (62/458), 47.6%(218/458), 10.3%(48/458) and 3.6%(16/442) of the cases are grouped under grade-3 thinness, grade -2 thinness, grade-1 thinness, normal, overweight and obese categories, respectively.

As their BMI value showed, 37.9%, 36.5%, 29.2%, 42.9%, 41.1 of malaria, malaria/hookworm, malaria/*A. lumbricoides*, malaria/*T. trichiura *and malaria/multiple intestinal helminth infected patients were in underweight (thin) status category, respectively. And the statistics for this difference is not significant. Similarly the statistics for underweight prevalence difference between malaria infection (37.9, 77/203) and malaria/helminth co infection in general (36.96, 95/257) is not significant (Table 3).

**Table 3:** Weight status of malaria and/or helminth infected cases at Alaba Health Center, southern Ethiopia, in November and December 2007

Infection type	Weight status						
	**Grade3 thinness %(N)**	**Grade 2 thinness %(N)**	**Grade 1 thinness %(N)**	**Normal %(N)**	**Overweight %(N)**	**Obese %(N)**	**Total (N)**

Malaria	18.7(38)	5(10)	14.3(29)	42.4(86)	15.8(32)	3.9(8)	203
Malaria/Hw	13.5(14)	6.7(7)	16.7(17)	50(52)	10.6(11)	2.9(3)	104
Malaria/Al	16.7(8)	4.2(2)	8.3(4)	64.6(31)	-	6.3(3)	48
Malaria/Tt	21.4(3)	21.4(3)	-	35.7(5)	21.4(3)	-	14
Malaria/mih	26(19)	2.7(2)	12.3(9)	56.2(41)	-	2.7(2)	73

Generally the prevalence of anaemia was higher in patients who were underweight (44.2%, 73/165) than those in the normal plus overweight (24.5%, 62/253) group. This difference was significant statistically (p < 0.001, Odds Ratio = 2.62, X^2 ^= 20.26). Similarly, high anaemia prevalence for malaria infection (X^2 ^= 12.29, p < 0.001, Odds Ratio = 2.15), malaria/hookworm infections (X^2 ^= 4.65, p = 0.031, Odds Ratio = 2.47) and malaria/*A. lumbricoides *infections (X^2 ^= 3.75, p = 0.05, Odds Ratio = 4.79) was observed in patients with underweight status when compared with those with normal and overweight status (Table 4). Particularly the highest proportion of anaemia for malaria/*A. lumbricoides *(X^2 ^= 3.85, p = 0.050) and malaria/hookworm(X^2 ^= 9.94, p = 0.041) infected patients *was *observed in those grouped under Grade-3 thinness. And the highest proportion of anaemia for malaria(X^2 ^= 14.8, p = 0.005) infected patients was observed in those grouped under Grade 1 thinness categories (Table 5).

**Table 4:** Results of the Mantel-Haenszel adjusted odds ratios of nutrition status and type of infection on anaemia for the confounding effect of age and sex.

Type of infection	% with underweight status cases who were anaemic(n/N)	% with normal and overweight status cases Who were anaemic (n/N)	Adjusted* Odds Ratio,(95%CI), x^2^(df), p-value	Over all Adjusted**Odds Ratio,(95%CI), x^2^(df), p-value
Malaria	36.4 (28/77)	16.1 (19/118)	2.98(1.51-5.85) 10.4(1),0.001	2.62(1.71-3.99) 20.26(1), p < 0.001
Malaria/Hw	55.3 (21/38)	33.3 (21/63)	2.47(1.08-5.65) 4.6(1),0.031	
Malaria/Al	62.5(5/8)	25.8(8/31)	4.79(0.93-24.75) 3.8(1),0.05	
Malaria/Tt	66.7(4/6)	0	NaN(NaN-NaN) NaN(1), p < 0.000	
Malaria/mih	50(15/30)	34.1(14/41)	1.93(0.73-5.06) 1.8(1),0.182	

* Within each cases the Odds were adjusted for both age and sex

** Within each cases the over all Odds were adjusted for both age and sex. For the combined data the odds were adjusted for cases, age and sex

**Table 5:** Distribution of anaemia by weight and infection status among the study participants at Alaba Kulito Health Center, SNNPR, South Ethiopia, Nov-Dec, 2007

Infection type	Anaemia					
	**Grade3 thinness %(n/N)**	**Grade 2 thinness %(n/N)**	**Grade 1 thinness %(n/N)**	**Normal %(n/N)**	**Overweight %(n/N)**	**Obese %(n/N)**

Malaria	36.8(14/38)	30(3/10)	37.9(11/29)	20.9(18/86)	3.1(1/32)	-
Malaria/Hw	71.4(10/14)	71.4(5/7)	35.3(6/17)	34.6(18/52)	27.3(3/11)	-
Malaria/Al	62.5(5/8)	-	-	25.8(8/31)	-	-
Malaria/Tt	100(3/3)	33.3(1/3)	-	-	-	-
Malaria/mih	52.6(10/19)	100(2/2)	33.3(3/9)	34.1(14/41)	-	-

## Discussion

Haematological abnormalities are considered a hallmark of malaria, especially in *Plasmodium falciparum *infection [25]. The present study also showed lower haemoglobin level in *P. falciparum *cases than those with *P. vivax*. A relatively lower mean haemoglobin concentration was also observed in those having mixed infections than those with single *Plasmodium *infected patients.

This study has demonstrated that cases with malaria/helminth co-infections had significantly higher prevalence of anaemia and lower mean haemoglobin concentration when compared with malaria infection cases. And this is in agreement with previous report [12], which observed a significant difference in haemoglobin concentration in malaria/helminth co-infected study patients and patients with malaria infection alone. Similarly this finding is comparable to another report [11], which showed the occurrence of low haemoglobin concentration in pregnant women co-infected with *Plasmodium *parasites and helminths in Kenya. In addition similar to previous reports [10] this study also indicated that anaemia prevalence and haemoglobin concentration difference between malaria/helminth co-infections and malaria infection shows an age and sex related pattern. In general these data are suggestive of an additive impact of helminth and malaria co-infection on aggravating anaemia [26,27] in children younger than 5 years and adults ≥ 15 years [10]. However, it should be noted that such associations may be confounded by socio-economic, genetic, and nutritional factors and that the effects of co-infection may vary by malaria and helminth transmission intensities [10]. Consequently, randomized controlled trials of combining malaria and helminth-specific interventions aimed at establishing the contribution of co-infection on anemia should be conducted in a range of transmission settings. The increased prevalence of anaemia in co infected cases may be attributed to chronic blood and iron loss due to worm infections in addition to the loss due to malaria. And we would suggest that combined intervention would be particularly relevant for vulnerable populations who are at the highest risk for anaemia. Thus, antihelminthic treatment could potentially be co-administered with malaria control to children younger than 5 years and adults ≥ 15 years in areas of seasonal malaria transmission.

Although the statistics for underweight prevalence difference is not significant between malaria infections and malaria/helminth co infections, more than one-third of the infected cases in both groups are undernourished. Several studies have shown that malaria has a negative effect on the nutritional status of under 5 children and individuals older than 5 years [27-29]. Malaria related symptoms, such as diarrhea and abdominal pain may lead to malabsorption of nutrients and decreased intake, respectively. In addition, proinflammatory cytokine such as TNF-α released against malaria antigens would adversely affect the nutritional status [30]. Soil-transmitted helminthiasis is also associated with varying degrees of malnutrition, the pathogenesis of which is poorly defined [7]. *Ascaris *and hookworm secret potent inhibitors of pancreatic enzymes, which may block host nutrient absorption in the small intestine directly [30]. Furthermore, soil-transmitted helminthiasis contributes to malnutrition by impairing appetite [31].

The high prevalence of anaemia in malaria patients of the underweight status groups than those in the normal weight and overweight groups suggests malnutrition as a risk factor for anaemia besides malaria and helminth infections. Anaemia prevalence was highest in the grade-3 thinness grouped subjects compared to those in other groups when the co-infection was malaria/*A*. *lumbricoides*. This seems to support the effect of *A. lumbricoides *on aggravating preexisting anaemia by decreasing appetite and thus food and iron intake [7].

As indicated above malaria and helminth co infections have impact on anaemia prevalence and weight status in infected patients. However other factors like diet and socio economic status which may have impact on haemoglobin levels and nutrition status are not considered in this study. And also, for each blood and stool specimens from participants only one slide was read to detect malaria and/or helminth infection and identify the type of species. In addition, there were small sample sizes in each infection types of different helminth intensity and *Plasmodium *parasitaemia level to make comparisons valid. Hence this study did not assess association between helminth intensity and *Plasmodium *parasiteamia with anaemia and weight status. These are some of the limitations of the study.

## Conclusion

Generally, malaria and soil-transmitted helminthiasis obviously contribute to anaemia and low weight status and these conditions are more pronounced in individuals concurrently infected with malaria and soil-transmitted helminths. Hence, simultaneous combat against the two parasitic infections is very crucial to improve health of the affected communities in economically developing countries.

## Competing interests

The authors declare that they have no competing interests.

## Authors' contributions

ML and AA conceived the project idea. ML, AD and AA with further input from BE designed the project. AD colleted the data and ML, and AA supervised data collection and assisted in the analysis. AD with the assistance of BE prepared the first draft of the paper and all authors reviewed and approved it.

## References

[B1] Keiser J N'Goran EK Traore M Lohourignon KL Singe RBH Lengeler C Tanner M Utzinger J Polyparasitism with *Schistosoma mansoni*, geohelminths, and intestinal protozoa in rural Côte d'Ivoire J Parasitol 2002 88 461 466 12099412 10.1645/0022-3395(2002)088[0461:PWSMGA]2.0.CO;2

[B2] Adrienne E Edridah M Jennifer K Clarkea K Pascal M Annette O Narcis B Kabatereineb R Simon B Epidemiology of helminth infection and their relationship to clinical malaria in Southwest Uganda Trans R Soc Trop Med Hyg 2005 99 18 24 10.1016/j.trstmh.2004.02.006 15550257

[B3] Mathieu N Worms and malaria; noisy nuisances and silent benefits Parasite Immunol 2002 24 7 391 401 10.1046/j.1365-3024.2002.00470.x 12164826

[B4] Kirsten E Alassane D Abdoulaye D Lansana S Abdoulaye K Drissa C Ando G Karim T Modibo D Issa D Marcelo B Christopher V Ogobara K Association of *Schistosoma haematobiumi *infection with protection against acute *Plasmodium falciparum *malaria in malaria children Am J Trop Med Hyg 2005 73 6 1124 1130 16354824 PMC2738948

[B5] Crawley J Reducing the burden of anaemia in infants and young children in malaria-endemic countries of Africa: from evidences to action Am J Trop Med Hyg 2004 71 25 34 15331816

[B6] McDevitt MA Xie J Gordeuk V Bucala R The anaemia of malaria infection: role of inflammatory cytokines Curr Hematol Rep 2004 3 97 106 14965485

[B7] Stephenson S Holland V Cooper S The public health significance of Trichuris trichiura Parasitol 2000 121 73 95 10.1017/S0031182000006867 11386693

[B8] Hotez JP Brooker S Bethony JM Bottazzi ME Loukas A Xiao S Hookworm Infect Eng J Med 2004 351 799 807 10.1056/NEJMra032492 15317893

[B9] Brooker S Clements ACA Hote PJ Hay SI Tatem AJ Bundy DA Snow RW Co-infection with hookworm and malaria is associated with lower haemoglobin levels and is common among African school children PLoS Med 2006 74 258 271

[B10] Akhwale WS Lum JK Kaneko A Eto H Obonyo C Bjorkman A Kobayakawa T Anemia and malaria at different altitudes in the western highlands of Kenya Acta Trop 2004 91 167 175 10.1016/j.actatropica.2004.02.010 15234666

[B11] Egwunyenga AO Ajayi JA Nmorsi OPG Duhlinska-Popova DD *Plasmodiuml *intestinal helminth Co- infections among pregnant Nigerian Women Mem inst Oswaldo Cruz Rio de Janeiro 2001 96 8 1055 1059 10.1590/s0074-02762001000800005 11784922

[B12] Nacher M Singhasivanon P Silachamroon U Treeprasertsuk S Krudsood S Gay F Mazier D Looareesuwan S Association of Helminth infection with decreased reticulocyte counts and hemoglobin concentration in Thai P. falciparum malaria Am J Trop Med Hyg 2001 65 4 335 337 11693879 10.4269/ajtmh.2001.65.335

[B13] Pullan R Brooker The health impact of polyparasitism in humans: are we Under-estimating the burden of parasitic diseases? Parasitol 2008 77 1 12 10.1017/S0031182008000346 PMC2645487 18371242

[B14] Crompton DW Nesheim MC Nutritional impact of intestinal helminthiasis during the human life cycle Ann Rev of Nut 2002 22 35 59 10.1146/annurev.nutr.22.120501.134539 12055337

[B15] Stephenson LS Latham MC Ottesen EA Malnutrition and parasitic helminth infections Parasitology 2000 121 23 38 10.1017/S0031182000006491 11386688

[B16] Nyakeriga AM Troye-Blomberg M Chematai AK Marsh K Williams TN Malaria and nutritional status in children living on the coast of Kenya Am J Clin Nuttr 2004 80 1604 1610 10.1093/ajcn/80.6.1604 15585775

[B17] Degarege A Animut A Legesse M Erko B Malaria severity status in patients with soil-transmitted helminth infections Acta Trop 2009 112 8 11 10.1016/j.actatropica.2009.05.019 19497286

[B18] WHO Physical status: the use and interpretation of anthropometry Geneva 1995 8594834

[B19] Cole TJ Bellizzi MC Flegal KM Dietz WH Establishing a standard definition for child overweight and obesity Int Surv BMJ 2000 320 1240 1243 10.1136/bmj.320.7244.1240 PMC27365 10797032

[B20] CDC BMI-for-age growth charts for girls and boys 2000 http://www.cdc.gov/growthcharts/Accessed on April 7, 2008

[B21] Cole TJ Flegal KM Nicholls D Body mass cut offs to define thinness in children and adolescents; international survey BMJ 2007 335 7612 194 215 10.1136/bmj.39238.399444.55 17591624 PMC1934447

[B22] Cheesbrough M Parasitological Tests, district laboratory practices in tropical countries 1998 Part I. England; Cambridge University press

[B23] WHO Basic Laboratory Methods in Medical Parasitology 1991 World Health Organization, Geneva

[B24] WHO Iron deficiency anaemia: assessment, prevention and control, a guide for programme managers 2001 World Health Organization, Geneva

[B25] Bashawri LAM Mandil AA Bahnassy AA Ahmed MA Malaria: Hematological aspects Annals of savil med 2002 22 373 377 10.5144/0256-4947.2002.372 17146269

[B26] Basavaraju SV PeterSchantz VMD Soil-transmitted helminths and Plasmodium falciparum malaria: epidemiology, clinical Manifestation, and the role of nitric oxide in malaria and geohelminth co-infection. Do worms have a protective role in P. falciparum? Nat Cent for Infec Diseas 2006 73 1099 1105 17285203

[B27] Brooker S Clements ACA Flote PJ Hay S Tatem AJ Bundy DAP Show RW The co-distribution of Plasmodium falciparum and hookworm among African school children J Mal Sci 2006 5 99 99 115 10.1186/1475-2875-5-99 PMC1635726 17083720

[B28] Snow RW Omumbo JA Lowe B Molyneux CS Obiero JO Palmer A Weber MW Pinder M Nahlen B Obonyo C Newbold C Gupta S Marsh K Relation between severe malaria morbidity in children and level of Plasmodium falciparum transmission in Africa Lancet 1997 349 1650 1654 10.1016/S0140-6736(97)02038-2 9186382

[B29] Friedman JF Kurtis JD Mtalib R Opollo M Lanar DE Duffy PE Malaria is related to decreased nutritional status among male adolescents and adults in the setting of intense perennial transmission J of Inf Disea 2003 188 449 457 10.1086/376596 12870128

[B30] Chu D Bungiro RD Ibanez M Molecular characterization of Ancylostoma ceylanicum Kunitz-type serine protease inhibitor Infect Immune 2004 74 2214 2221 10.1128/IAI.72.4.2214-2221.2004 PMC375216 15039345

[B31] Michael C Global health impact of soil-transmitted nematodes The Pedt Inf Disea J 2004 23 663 664 10.1097/01.inf.0000132228.00778.e4 15247606

